# Localized IRES-Dependent Translation of ER Chaperone Protein mRNA in Sensory Axons

**DOI:** 10.1371/journal.pone.0040788

**Published:** 2012-07-24

**Authors:** Almudena Pacheco, Jeffery L. Twiss

**Affiliations:** Department of Biology, Drexel University, Philadelphia, Pennsylvania, United States of America; University of Louisville, United States of America

## Abstract

Transport of neuronal mRNAs into distal nerve terminals and growth cones allows axonal processes to generate proteins autonomous from the cell body. While the mechanisms for targeting mRNAs for transport into axons has received much attention, how specificity is provided to the localized translational apparatus remains largely unknown. In other cellular systems, protein synthesis can be regulated by both cap-dependent and cap-independent mechanisms. The possibility that these mechanisms are used by axons has not been tested. Here, we have used expression constructs encoding axonally targeted bicistronic reporter mRNAs to determine if sensory axons can translate mRNAs through cap-independent mechanisms. Our data show that the well-defined IRES element of encephalomyocarditis virus (EMCV) can drive internal translational initiation of a bicistronic reporter mRNA in distal DRG axons. To test the potential for cap-independent translation of cellular mRNAs, we asked if calreticulin or grp78/BiP mRNA 5′UTRs might have IRES activity in axons. Only grp78/BiP mRNA 5′UTR showed clear IRES activity in axons when placed between the open reading frames of diffusion limited fluorescent reporters. Indeed, calreticulin’s 5′UTR provided an excellent control for potential read through by ribosomes, since there was no evidence of internal initiation when this UTR was placed between reporter ORFs in a bicistronic mRNA. This study shows that axons have the capacity to translate through internal ribosome entry sites, but a simple binary choice between cap-dependent and cap-independent translation cannot explain the specificity for translation of individual mRNAs in distal axons.

## Introduction

Eukaryotic cells can temporally and spatially regulate protein composition of subcellular domains through translation of mRNAs transported to these sites. This is particularly relevant to neurons where both the post-synaptic and pre-synaptic processes can be separated from the cell body by long distances. Initial studies suggested that localized protein synthesis in neurons is restricted to dendrites. However, several different laboratories have demonstrated that axons contain ribosomes, translation factors and mRNAs, and are capable of generating proteins when isolated from the cell body (for review see [1]). Nevertheless, little is known about the mechanisms that are used to bring specificity to the axon’s protein synthesis apparatus.

It is appealing to hypothesize that axons maintain multiple levels of translational regulation to temporally match the synthesis of new proteins to the physiological needs of this subcellular domain. RNA profiles of axons and dendrites have shown that an increasingly complex fraction of the neuron’s transcriptome can localize into these processes [2], [3]. The 3′UTRs of mRNAs have most often been linked to subcellular localizing activity, including localization into axons [4]. Localization of the mRNAs is driven by RNA binding proteins that recognize *cis*-elements in the mRNAs [4]. For some localizing mRNAs, the same RNA binding protein that is needed for the mRNA’s transport to subcellular sites regulates translation. For example, ZBP1 inhibits translation of its cargo mRNAs and this translational repression is released upon phosphorylation of ZBP1 [5]. Some guidance cues have been shown to trigger phosphorylation of ZBP1 in axons, presumably allowing stimulus-dependent translation of the ZBP1 mRNA cargo [6]. For other axonal mRNAs, the mechanisms for translational silencing are unknown, and there are now several examples of localized mRNAs that are stored until needed. Importin β1, RanBP1, and vimentin mRNAs are stored in axons until needed, with an axotomy-induced increase in Ca^2+^ levels activating their translation [7]–[9]. Though the molecular mechanisms are still unclear, these observations indicate that mRNA transport and local translational activation of the mRNA are not always mechanistically linked.

Initiation of translation is the rate-limiting step of protein synthesis in eukaryotes. Initiating translation requires a set of specialized proteins known as initiation factors (eIFs) that recruit the 40S ribosome subunit to the m^7^GpppN structure or m^7^GTP residue (or ‘cap’) located at the immediate 5′-end of most eukaryotic mRNAs [10]. The cap-dependent initiation complex then scans the 5′-untranslated regions (UTR) until finding an AUG initiation codon in the appropriate context to start protein synthesis [11]. In contrast to the general cap-dependent mechanism of protein synthesis, some viral RNAs have been shown to initiate translation through an alternate mechanism driven by internal ribosome entry sites (IRES) [12], [13]. These *cis*-acting 5′UTR elements form secondary and tertiary RNA structures that can recruit the translational machinery to an internal position in the mRNA. This allows the ribosome to bypass stable RNA structures in the 5′ UTRs and internally initiate translation of the mRNA [14]. This has also been shown to occur for some cellular mRNAs, and cap-independent protein synthesis can provide a selective advantage for generating new proteins under conditions when traditional cap-dependent translation is compromised [15]. Cap-independent mRNA translation has been demonstrated in dendrites [16], [17], but the possibility that cellular mRNAs transported into axons from the cell body can be translated through internal initiation of translation has not been tested.

We previously showed that calreticulin mRNA contains two 3′UTR *cis*-elements that confer subcellular localization; a proximal RNA element for stimulus-dependent transport requiring activation of c-Jun N-terminal kinase (JNK) pathways and a second more distal element for constitutive transport into axons [18]. The 5′UTR of calreticulin confers translational regulation in response to lysophosphatidic acid (LPA) through phosphorylation of eIF2α [19]. Thus, the axonal transport and localized translation of calreticulin mRNA can be through distinct mechanisms. Here we show that, despite this activation of axonal calreticulin mRNA translation upon inhibition of eIF2α, calreticulin’s 5′UTR has no IRES activity in sensory neurons. However, axonal processes are capable of cap-independent translational regulation since both viral RNA and grp78/BiP mRNA 5′UTRs function as IRES’s when placed between cistrons of axonally targeted bicistronic reporter mRNAs.

## Results

**Figure 1:**
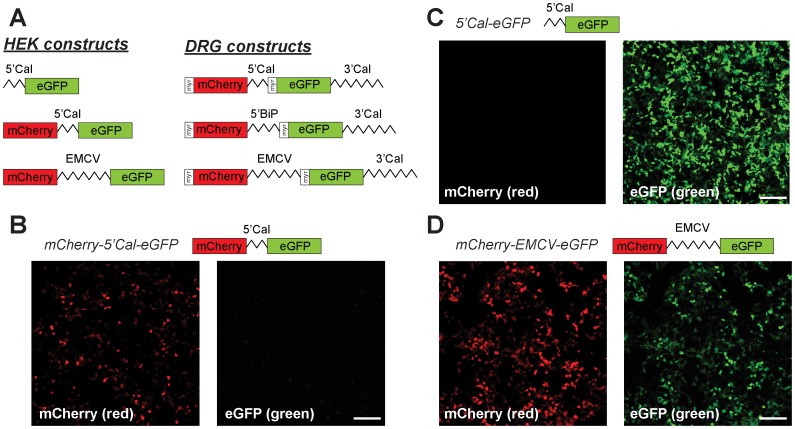
5′UTR of calreticulin mRNA does not show IRES activity in HEK-293 cells. A , Schematic of monocistronic and bicistronic constructs used for mRNA expression in HEK cells and DRG neurons is shown. 5′CAL and 3′CAL correspond to 5′ and 3′UTRs of rat calreticulin mRNA. 5′BiP corresponds to the 5′UTR of grp78/BiP mRNA. EMCV corresponds to the 5′ leader sequence of the encephalomyocarditis virus RNA. The constructs used in HEK cells contained standard mCherry and eGFP, while those for neuronal transfections contained diffusion limited mCherry^myr^ and eGFP^myr^ reporters. The neuronal constructs also included an axonal targeting 3′UTR (3′Cal). **B-D**, Representative exposure matched fluorescent images are shown for HEK-293 cells, 48 h post-transfection, for bicistronic pmCherry-5′CAL-eGFP (**B**), monocistronic p5′CAL-eGFP (**C**) and bicistronic pmCherry-EMCV-eGFP (**D**) [scale bars  = 200 µm].

We previously showed that the mRNA encoding the ER chaperone protein calreticulin localizes to the axons of DRG and cortical neurons through *cis*-elements in its 3′UTR [18]. Synthesis of calreticulin protein is increased by release of ER Ca^2+^ stores in isolated sensory axons [20]. This translational control of axonal calreticulin mRNA is conveyed by its 5′UTR [19]. The 5′UTR of rat calreticulin shows high primary sequence identity between vertebrates, but it is short (63 nucleotides) and has a single AUG initiation codon. Despite this relatively short length, this 5′UTR is a GC-rich sequence (68%) that is predicted to form a stem-loop secondary structure. Considering these features and the translational regulatory activity of calreticulin’s 5′UTR [19], we asked if the 5′UTR of calreticulin might have IRES activity. For this, we generated bicistronic fluorescent reporter constructs placing the calreticulin 5′UTR in the intercistronic region between mCherry and eGFP cDNAs (Fig. 1A). This generates a reporter mRNA where mCherry is expressed through cap-dependent translation and, if expressed, eGFP must be translated through recruitment of ribosomes internal to the 5′UTR. We initially examined expression in HEK cells that can be transfected at high efficiency. mCherry fluorescence from the 5′ open reading frame (ORF) was clearly visible, but no GFP protein signal was detected from the 3′ eGFP cistron (Fig. 1B). However, robust GFP signals were seen when the 5′UTR of calreticulin was placed upstream of a monocistronic reporter construct (Fig. 1C). Consistent with previous studies of the EMCV IRES element in HEK cells [21], a bicistronic reporter construct with EMCV IRES inserted between mCherry and eGFP ORFs showed robust GFP fluorescence in the transfected HEK cells (Fig. 1D). Thus, the bicistronic strategy used here can provide for cap-independent translation through EMCV IRES, but the 5′UTR of calreticulin appears to lack any IRES activity in the HEK cells.

**Figure 2:**
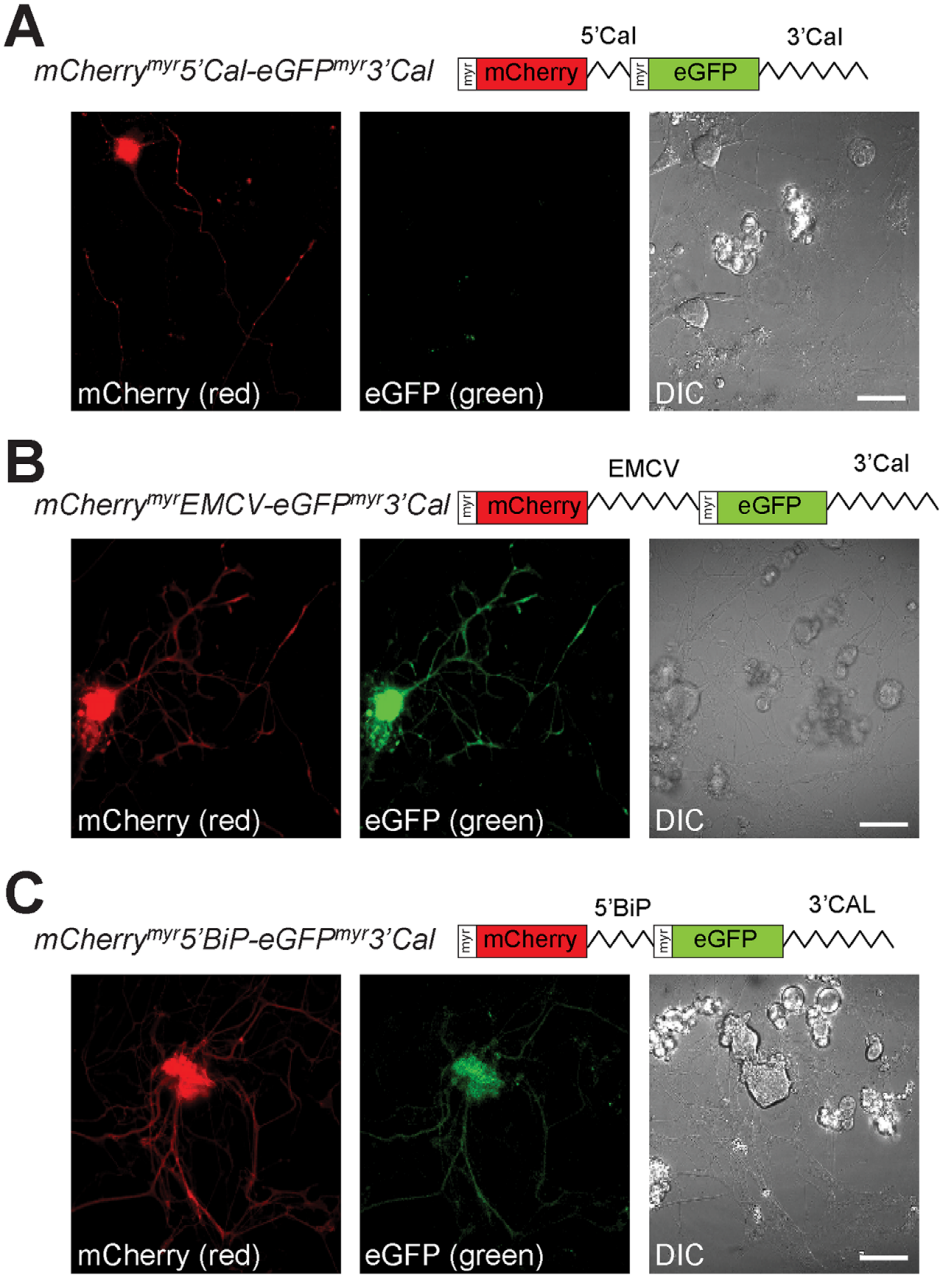
Bicistronic reporters support IRES-dependent translation in sensory neurons. Representative static images of DRG neurons transfected with bicistronic pmCherry^myr^5′Cal-eGFP^myr^3′Cal (**A**), pmCherry^myr^EMCV-eGFP^myr^3′Cal (**B**), and pmCherry^myr^5′BiP-eGFP^myr^3′Cal (**C**) reporters are shown at 48 h post-transfection. Both mCherry (red, left panel) and eGFP (green, right panel) fluorescence is seen in the cell bodies and axons of mCherry^myr^EMCV-eGFP^myr^3′Cal and mCherry^myr^5′BiP-eGFP^myr^3′Cal expressing neurons (**B,C**), only the mCherry signals are seen for the mCherry^myr^5′Cal-eGFP^myr^-3′Cal expressing neurons (**A**). These data suggest that the 5′UTR of grp78/BiP mRNA but not calreticulin’s 5′UTR can function as an IRES in sensory neurons [scale bars  = 50 µm].

**Figure 3:**
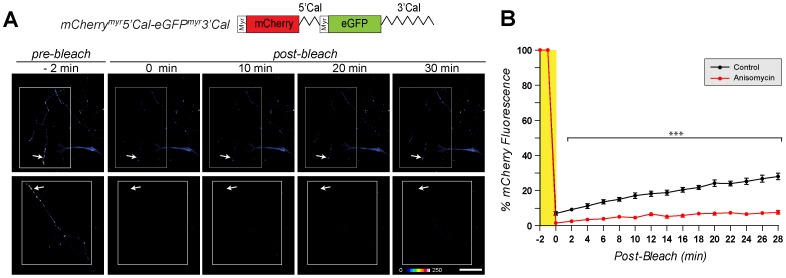
5′UTR of calreticulin does not support cap-independent translation in sensory neurons. A , Representative time-lapse sequences from FRAP analyses of DRG neurons transfected with pmCherry^myr^5′Cal-eGFP^myr^3′Cal are shown. mCherry fluorescence is displayed as a spectrum with white being the brightest signal as indicated. The white boxed regions represent the regions subjected to photobleaching and the arrows indicate the regions of the terminal axon where recovery was quantified. The upper row shows cultures standard medium and lower row shows cultures pretreated with 150 µM anisomycin [scale bar  = 50 µm]. **B**, Quantification of normalized mCherry fluorescence intensity in ROI as indicated from multiple FRAP sequences are shown. Signals are shown as the average percent of pre-bleach levels ± SEM (n ≥12 over at least 4 independent transfections; *** =  p≤0.001 by two-way ANOVA comparing conditions to t = 0 min for indicated time points).

IRES-mediated translation depends on availability of IRES trans-acting factors (ITAFs) that modulate IRES activity [22], [23]. Consequently, activity of individual IRES elements can vary considerably from one cell type to another [24], [25]. In a recent study, we saw differential translational regulation of calreticulin mRNA in axons compared to neuronal cell bodies [19]. Thus, we asked if the 5′UTR of calreticulin might have IRES activity in axons. For this, we generated a bicistronic vector with the diffusion-limited reporters mCherry^myr^ and eGFP^myr^ to visualize sites of protein synthesis in neuronal processes (Fig. 1A) and the 3′UTR of calreticulin mRNA for axonal targeting [18]. Similar to the HEK cells, the 5′UTR of calreticulin showed no evidence of IRES activity when placed between cistrons of the axonally targeted bicistronic mCherry^myr^5′Cal-eGFP^myr^3′Cal mRNA. Although mCherry signals were clearly visible in the axons and cell body of the DRG neurons, neither the axons nor cell body showed any GFP fluorescence (Fig. 2A). FRAP analyses showed significant recovery of the axonal mCherry fluorescence after photobleaching that was prevented by pretreatment with translational inhibitors (Fig. 3; Videos S1, S2). Thus, the mCherry^myr^5′Cal-eGFP^myr^-3′Cal construct generates an mRNA that can be used to locally synthesize mCherry^myr^ through cap-dependent translation but does not support cap-independent translation of the downstream eGFP^myr^ cistron.

**Figure 4:**
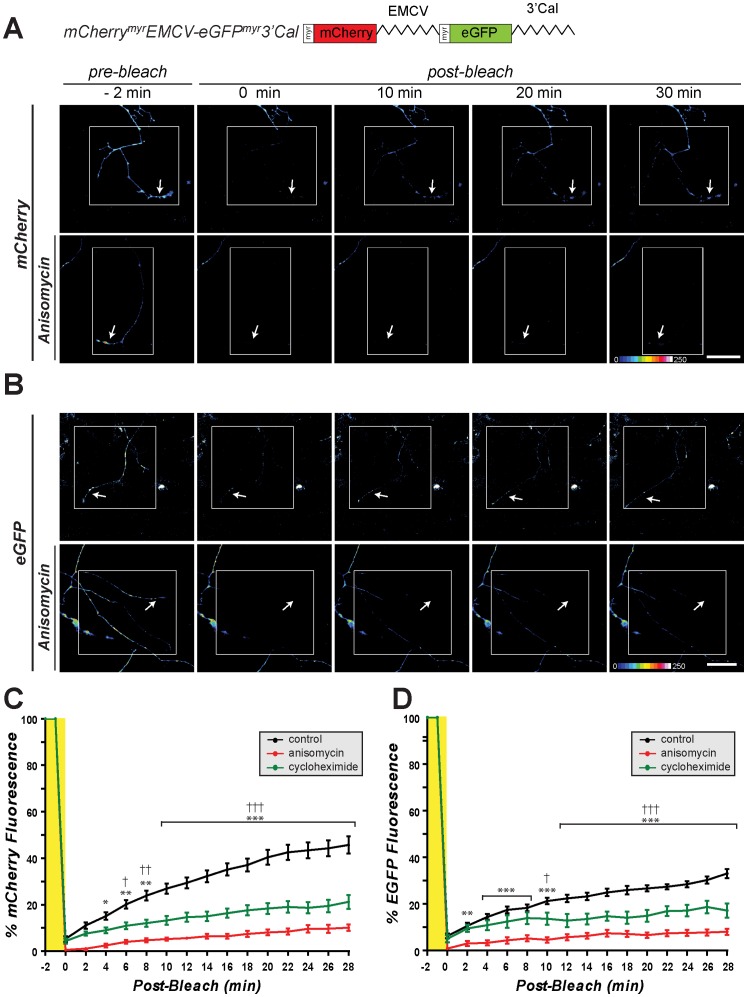
EMCV IRES supports cap-independent translation in axons. A-B , Representative time-lapse sequences from FRAP analyses of DRG neurons transfected with pmCherry^myr^EMCV-eGFP^myr^3′Cal as in Fig. 3 are shown. mCherry and eGFP signals are displayed as a spectrum with white being the brightest signal as indicated. The white boxed regions represent the regions subjected to photobleaching and the arrows indicate the regions of the terminal axon where recovery was quantified. Sequences for cap-dependent translation of mCherry are shown in **A** and for IRES-dependent translation of eGFP are shown in **B**. The upper rows for A and B show cultures standard medium and lower rows show cultures pretreated with 150 µM anisomycin [scale bar  = 50 µm]. **C-D**, Quantifications of axonal mCherry and eGFP signals from multiple FRAP sequences from DRG neurons transfected with mCherry^myr^EMCV-eGFP^myr^-3′Cal are shown. Signals in each individual series are normalized to pre-bleach levels and are expressed as average percent prebleach signals ± SEM (n *≥*8 over at least 4 independent transfections; *p≤0.05, **p*≤*0.01, and ***p*≤*0.001 for control vs. anisomycin time points and †p≤0.05, ††p*≤*0.01, and †††p≤0.001 for control vs. cycloheximide time points by two-way ANOVA compared to t  = 0 min post-bleach). Both mCherry and eGFP fluorescence in distal axons shows recovery after photobleaching that is attenuated by protein synthesis inhibitors. These data indicate that the EMCV IRES can drive internal translational initiation in axons.

Although IRES-dependent translation has been documented in dendrites [17], axonal processes have never been tested for this ability. To address the question of IRES-dependent translation in axons, we initially asked if the well-characterized EMCV IRES could support internal translational initiation in axons. For this, we substituted the EMCV IRES sequence for the calreticulin 5′UTR in pmCherry^myr^5′Cal-eGFP^myr^-3′Cal to generate a bicistronic construct encoding mCherry^myr^EMCV-eGFP^myr^3′Cal mRNA. In contrast to pmCherry^myr^5′Cal-eGFP^myr^3′Cal transfections, eGFP fluorescence was clearly visible in the cell bodies and axons of DRG neurons expressing mCherry^myr^EMCV-eGFP^myr^3′Cal mRNA (Fig. 2B). FRAP analyses of distal axons showed that the axonal mCherry and eGFP fluorescence recovered from photobleaching (Fig. 4A; Video S3). Moreover, recovery of the mCherry and eGFP fluorescence was attenuated by anisomycin and cycloheximide (Fig. 4B, Video S4). Since these agents block distinct steps of translational elongation [26], [27], these data emphasize that the recovery of eGFP fluorescence in the axons is undoubtedly through internally initiated translation of the axonal mCherry^myr^EMCV-eGFP^myr^3′Cal mRNA. Thus, it appears that sensory axons tested here do have the ability to internally initiate translation through the EMCV IRES element.

**Figure 5:**
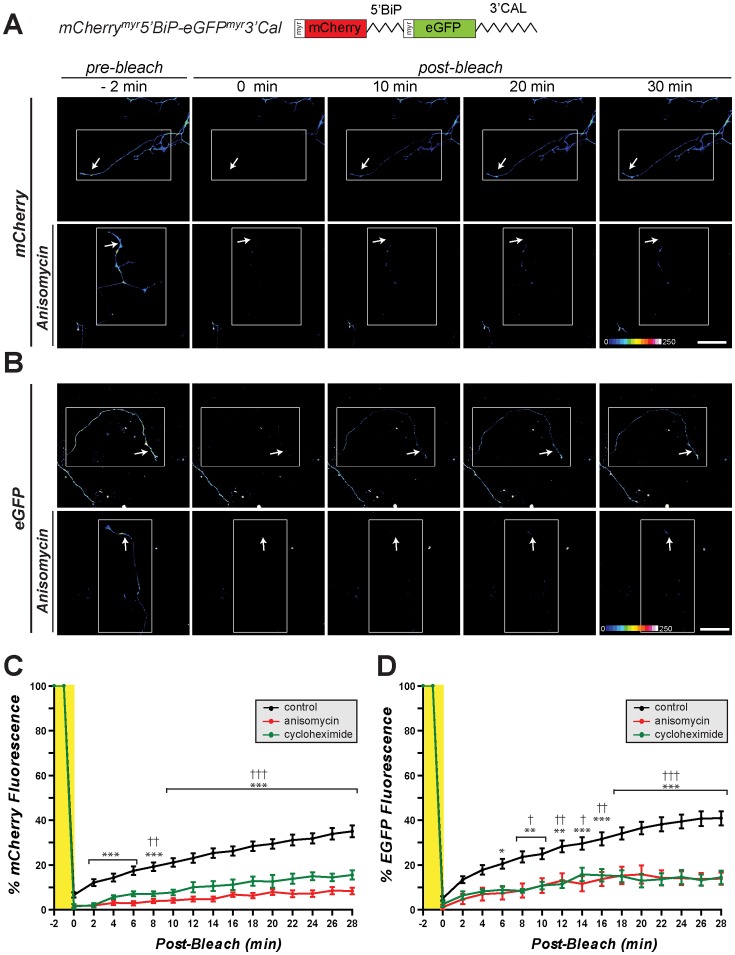
5′UTR of grp78/BiP mRNA can function as an IRES in axons. A-B , Representative time-lapse sequences from FRAP analyses of DRG neurons transfected with pmCherry^myr^5′BiP-eGFP^myr^3′Cal as in Fig. 4 are shown. Sequences for cap-dependent translation of mCherry are shown in **A** and for IRES-dependent translation of eGFP are shown in **B**. The upper rows for A and B show cultures standard medium and lower rows show cultures pretreated with 150 µM anisomycin [scale bar  = 50 µm]. **C-D**, Quantifications of axonal mCherry and eGFP signals from multiple FRAP sequences from DRG neurons transfected with mCherry^myr^5′BiP-eGFP^myr^-3′Cal are shown. Signals in each individual series are normalized to pre-bleach levels and are expressed as average percent prebleach signals ± SEM (n *≥*6 over at least 4 independent transfections; *p≤0.05, **p*≤*0.01, and ***p*≤*0.001 for control vs. anisomycin time points and †p≤0.05, ††p*≤*0.01, and †††p≤0.001 for control vs. cycloheximide time points by two-way ANOVA compared to t  = 0 min post-bleach). Both mCherry and eGFP fluorescence in distal axons shows recovery after photobleaching that is attenuated by protein synthesis inhibitors. These data indicate that the 5′UTR of rat grp78/BiP mRNA can drive IRES-dependent translation in axons.

Since the DRG neurons showed capacity for internal initiation of a viral RNA IRES element but not of the calreticulin’s mRNA’s 5′UTR, we asked if other cellular mRNAs might show IRES activity in the axonal compartment. grp78/BiP was the first cellular mRNA demonstrated to have cap-independent translation [28]. Similar to calreticulin mRNA, grp78/BiP mRNA encodes an ER chaperone protein that is locally translated in sensory axons [29], [30]. Thus, we generated an axonally targeting bicistronic construct with the 5′UTR of rat grp78/BiP in the intercistronic region of mCherry^myr^ and eGFP^myr^ (mCherry^myr^5′BiP-eGFP^myr^3′Cal) to test for IRES activity in the DRG axons. Robust mCherry and eGFP signals were seen in the cell body and axons of mCherry^myr^5′BiP-eGFP^myr^3′Cal expressing neurons (Fig. 2C). Both mCherry and eGFP fluorescence in the distal axons of mCherry^myr^-5′BiP-eGFP^myr^-3′Cal transfected neurons recovered from photobleaching over a time course paralleling what we have previously demonstrated for axonally generated reporter proteins (Fig. 5; Video S5) [9], [18]. This recovery of axonal reporter fluorescence after photobleaching was also blocked by anisomycin and cycloheximide (Fig. 5B; Video S6). Thus, the axonal compartment of the adult rodent sensory neurons used here has the capacity to internally initiate translation through BiP IRES element but not through calreticulin 5′UTR. We recently showed that the 5′UTR of calreticulin mRNA confers local translational activation by lysophosphatidic acid (LPA) [19]. However, the mCherry^myr^5′Cal-GFP^myr^3′Cal expressing neurons did not show any detected GFP fluorescence, neither in axons or cell body, after 2 hours LPA treatment (Fig. S1).

## Discussion

Localized synthesis of neuronal proteins contributes to axonal responses to guidance cues and injury [31]. Several lines of evidence suggest that the axonal translational machinery can match the translation of different mRNAs to the stimulus provided. For example, positive and negative guidance cues have been linked to translation of different axonal mRNAs [32]–[34]. Additionally, nerve injury triggers translation of axonal mRNAs whose encoded proteins are needed to stimulate transcriptional responses in the neuronal cell body [7]–[9], [35]. Recent work has shown that phosphorylation of the RNA binding protein ZBP1 regulates the translation of specific axonal mRNAs [6], [36]. However, only a few axonal mRNAs are known targets of ZBP1 and there are likely to be many axonal mRNAs that are not regulated by ZBP1. Consistent with this, we recently showed that calreticulin mRNA studied here is not a target for ZBP1 binding in axons [36]. With hundreds of other mRNAs localizing into axons, it is likely that axons have multiple mechanisms at their disposal for temporally regulating translation of different axonal mRNA cohorts.

The data shown here indicate that at least some axonal mRNAs can be translated using internal initiation elements. It is likely that other polarized cells that make use of localized protein synthesis will utilize cap-independent translation to locally regulate synthesis of proteins.

Translation through IRES elements was initially recognized in viral RNAs, where viral proteins inactivate cap-dependent translation favoring translation of viral RNAs without the need for the eIF4 cap-binding complex [12], [13]. Cap-dependent translation can be down regulated during development, stress, and disease states such that internal initiation is needed for continued protein expression [37], [38]. This brings a level of specificity to the translational apparatus to preferentially generate proteins needed under unique physiological states. Using FRAP to detect axonally translated reporter proteins, our studies show that both viral and cellular RNA elements previously shown to have IRES activity in other cellular systems provide internal initiation of translation in sensory axons. In previous FRAP studies focusing on *cis*-elements for axonal RNA transport, both isolated axons and repetitive photobleaching of proximal axonal segments was used to rule out the possibility that cell body-derived reporter protein accounted for the rapid recovery of fluorescence [9]. In our hands, this recovery from photobleaching in distal axons has consistently correlated with axonal localization of the reporter mRNAs [18], [35]. In contrast, we are able to fully differentiate RNA transport and translational control with the bicistronic reporters used here, since the neurons expressing axonally targeted mCherry^myr^5′Cal-eGFP^myr^3′Cal mRNA showed recovery of mCherry.

The EMCV IRES used here is a highly structured element presents in the 5′UTR region of its positive single strand RNA [12]. This RNA structure is commonly used in commercial bicistronic vectors [39]. Activity of the EMCV IRES in the sensory axons shown here may have important implications for effects of viral infection on mature neurons. For example, IRES of other picornaviruses such as poliovirus can contribute to the neurovirulence of different viruses [40]. Recently, the Jaffrey group showed evidence for IRES driven translation through EMCV IRES with static imaging approaches applied to axons transduced with a modified Sindbis RNA virus [41]. Our study advances their observations by showing that a bicistronic mRNA can be transported into axons from the cell body and locally translated through cap-independent mechanisms. Moreover, we show that an axonally transported cellular mRNA, grp78/BiP, has localized IRES activity in axons. The 5′UTR of grp78/BiP has similarly been shown to have IRES activity in other cellular systems [28], [42]. Heat shock was shown to drive translation through the 5′UTR of this Ca^2+^-binding ER chaperone protein [21]. Both transcription and translation of grp78/BiP and other chaperone proteins has been shown to be increased with other forms of cellular stress that lead to the unfolded protein response [43]. We have previously shown that the 3′UTR of grp78/BiP mRNA is sufficient for its transport into axons [18]. Although it is not clear what cellular mechanisms trigger cap-independent translation of this mRNA, our data suggest that grp78/BiP mRNA translation in axons can be regulated through RNA element(s) distinct from those used for targeting the mRNA for transport into axons. Local siRNA-mediated depletion of mRNAs from axons of cultured neurons and in peripheral nerve in vivo shows that axons have RNA interference machinery [44], [45]. Moreover, microRNAs were detected in sympathetic axons in culture and shown to block translation of axonal CoxIV mRNA [46], [47]. Thus, non-coding mRNAs may offer a means for translational specificity beyond the choice between cap-dependent and IRES-mediated translation that we have tested here.

In summary, we show that a bicistronic mRNA can be transported into the axonal compartment and locally translated through cap-dependent and cap-independent mechanisms. This provides evidence for multiple mechanisms of translational control for axonal mRNAs. Metabolic labeling of axons indicated that only 5–10% of total cellular protein synthesis occurs in the axons [48], [49]. For the cap-independent translation seen here, both viral and cellular RNAs are functional IRES elements in axons similar to what has been documented for studies in whole cells. Efficiency of different IRES elements has been reported to be cell type specific possibly due to the availability of cellular factors required for a particular IRES [22], [50]. Further studies will be needed to understand how IRES-dependent translation is mediated in axons. Nonetheless, our studies indicate that distinct mechanisms have evolved to modulate translation of axonal localized mRNAs.

## Materials and Methods

### Cell Culture and Transfections

HEK-293 cells were maintained in 10% fetal bovine serum supplemented DMEM at 37°C and 5% CO_2_ on plastic dishes. HEK-293 cells were transfected with 2 µg plasmid using Lipofectamine 2000 Reagent per manufacturer’s instructions (Invitrogen). Transfected cells were cultured for 48 h on glass coverslips.

Primary cultures of L4-5 dorsal root ganglion (DRG) were prepared from adult Sprague Dawley rats (175 g). These were dissociated using 36 mg/ml collagenase type XI (Sigma) for 30 min. The dissociated DRGs were transfected using *AMAXA Nucleofector* apparatus with the *SCN Nucleofector kit* (program G-8; Lonza, Inc.). Cells were then resuspended in DMEM/F12 (Mediatech) with 10% horse serum (Hyclone) and cultured for 48–72 h. For DRGs, medium was replaced at 20 h post-transfection and 10 µM arabinofuranosyl cytidine (Sigma) was included to decrease proliferation of non-neuronal cells.

### DNA Expression Constructs

All constructs used for expression in HEK cells were generated from the pBMN vector backbone (provided by Dr. Luis Sigal). p5′Cal-mCherry^myr^3′Cal was used as a template for PCR to generate 5′UTR rat calreticulin (GenBank Accession, NM_022399) with Not1 and Nco1 restriction sites for subcloning upstream of the eGFP coding sequence in pBMN. This generated the monocistronic construct p5′CAL-eGFP. mCherry was then amplified from p5′Cal-mCherry^myr^3′Cal incorporating BamH1 and Not1 restriction sites for subcloning upstream of the above 5′UTR calreticulin to generate the bicistronic construct pmCherry-5′CAL-eGFP. The same mCherry PCR product was cloned upstream of the EMCV IRES sequence (NC_001479; IRESite Id: 140) in the pBMN vector to generate the bicistronic construct pmCherry-EMCV-eGFP (see Fig. 1A). In each case where PCR products were used for this and subsequent vector production, inserts were fully verified by sequencing.

All the bicistronic constructs for testing axonal translation in DRG neurons were generated in the pcDNA3.1 backbone (Invitrogen). For these, we used eGFP and mCherry with an N-terminal myristylation signals (eGFP^myr^ and mCherry^myr^, respectively). The original eGFP^myr^ vector with 5′ and 3′ UTRs of CamKIIα was obtained from Dr. Erin Schuman [51]; the mCherry^myr^ was generated by PCR [19]. The diffusion-limited bicistronic vectors were prepared in two consecutive steps. First, a monocistronic construct pcDNA3.1-mCherry^myr^ was generated by cloning BamH1/Not1 fragment of mCherry^myr^ from p5′Cal-mCherry^myr^-3′Cal construct [19] into pcDNA3.1. 5′Cal-eGFP^myr^3′Cal insert was then isolated by EcoRV/Xho1 digest of p5′Cal-eGFP^myr^3′Cal and subcloned downstream of mCherry^myr^ in pCDNA3.1-mCherry^myr^ to yield the bicistronic construct pmCherry^myr^5′Cal-eGFP^Myr^3′Cal (Fig. 1A).

For the EMCV IRES and grp78/BiP 5′UTR bicistronic constructs, an intermediate pmCherry^myr^-eGFP^myr^3′Cal was generated such that EcoR1 and Xho1 restriction sites were inserted for cloning purposes between the mCherry^myr^ and eGFP^myr^ open reading frames. cDNA for the EMCV IRES (NC_001479; IRESite Id: 140) was generated by PCR from pBMN backbone. EcoR1/Xho1 digested EMCV IRES PCR product was subcloned to generate pmCherry^myr^EMCV-GFP^myr^3′Cal. Then grp78/BiP IRES insert (GenBank Accession X87949.1; IRESsite Id: 570) was isolated from an EcoRV/Xho1 digest of pUC57-BiP (GenScript) and subcloned between the mCherry^myr^ and eGFP^myr^ cistrons to generate pmCherry^myr^5′BiP-eGFP^Myr^3′Cal (Fig. 1A).

### Static and Live Cell Imaging

Expression of mCherry and eGFP was initially tested in HEK-293 cells by static imaging. For this, transfected cultures were fixed in buffered 4% paraformaldehyde and coverslips were mounted in *Prolong Gold Anti-fade* (Invitrogen). These samples were examined using a *10X Plan Apochromat* objective (0.40 NA) on *FluoView 1000* confocal microscope (Olympus).

For live-cell imaging, the DRG cultures were imaged directly at 48–72 hours after transfection using Zeiss *LSM700* confocal microscope fitted with an enclosure to maintain 5% CO_2_ and 37°C. To test for localized translation, we used fluorescence recovery after photobleaching (FRAP) as previously described with minor modifications. 40X *C-Apochromat-Korr M27* water immersion objective (1.20 NA) was used for imaging, and the confocal pinhole was set to 4 airy units to ensure that the entire 2–4 µm thickness of axons was fully photobleached. DIC imaging was used to visualize neuronal processes; these are easily distinguised from Schwann cells in these cultures by presence of a terminal growth cone and length of several hundred microns. This was facilitated by the use of low density cultures for these imaging studies. Prior to photobleaching, neurons were imaged every 60 sec. for 2 min. to establish baseline intensity using 488 and 555 nm lasers at 6.0 and 0.5% power, respectively. For photobleaching, the terminal ≥150 µm of distal axon was exposed to 100% laser power at 488 and/or 555 nm (for eGFP and mCherry, respectively) to reach a pixel intensity of <5% of the prebleach signals (50 iterations). Recovery of mCherry and eGFP was then monitored in a region of interest (ROI) comprising the terminal 50 µm of the axons every 60 sec. over 30 min. using 488 nm laser at 0.5% power and 555 nm laser at 6.0% power. To test for translation-dependent recovery, cultures were pretreated with 150 µM anisomycin or 150 µg/ml cycloheximide for 30 min. prior to photobleaching. For treatment with LPA, transfected cultures were imaged before and after 2 hour exposure to 30 µM LPA (BioMol) that was diluted in 0.1% bovine serum albumin (BSA). Control for this experiment consisted of cultures treated with 0.1% BSA.

Images were analyzed using *ZEN 2010* software package (Zeiss) to calculate the mean fluorescence intensity within the ROI. Fluorescent intensity at each time point was normalized to the average pre-bleach intensity. Each construct was analyzed in ≥6 neurons over at least 4 independent transfections/culture preparations.

### Statistical Analyses

Data were analyzed using *GraphPad Prism 5* software package. Two-way ANOVA followed by Bonferroni post-hoc multiple comparisons were used to compare the time for the recovery between treatments at each time point. All values were expressed as mean ± standard error of the mean (SEM). P values of ≤0.05 were considered as significant.

## Supporting Information

Figure S1 **LPA effect in 5′UTR cap-independent translation.** Representative images of DRG neurons expressing indicated bicistronic mRNAs are shown after 2 h exposure to 30 µM LPA. Only the mCherry signal is seen for the mCherry^myr^5′Cal-eGFP^myr^-3′Cal expressing neurons, both in cell body (arrowhead) and axons (arrow). These data indicate LPA does not trigger cap-independent translation through calreticulin’s 5′UTR in sensory neurons [scale bars  = 50 µm].
(TIF)

Video S1 **Recovery of axonal mCherry fluorescence in mCherry^myr^5′Cal-eGFP^myr^ mRNA expressing DRG neurons.** Representative FRAP sequence of a neuron transfected with pmCherry^myr^5′Cal-eGFP^myr^3′Cal is shown with fluorescent intensity shown as a spectrum as outlined in Figure 3 (original time lapse  = 30 min, with 2 min pre-bleach and 28 min post-bleach at 1 frame/min) [scale bar  = 50 µm].
(MOV)

Video S2 **Protein synthesis inhibition attenuates axonal recovery of cap-dependent translation in mCherry^myr^5′Cal-eGFP^myr^ mRNA expressing DRG neurons.** Representative FRAP sequence as in Videos S1 except cultures were pretreated with 150 µM anisomycin prior to photobleaching (original time lapse  = 30 min, with 2 min pre-bleach and 28 min post-bleach at 1 frame/min) [scale bar  = 50 µm].
(MOV)

Video S3 **Recovery of axonal eGFP fluorescence in mCherry^myr^EMCV-eGFP^myr^ mRNA expressing DRG neurons.** Representative FRAP sequence of a neuron transfected with pmCherry^myr^EMCV-eGFP^myr^3′Cal is shown as outlined in Figure 4 with fluorescent intensity shown as a spectrum as indicted (original time lapse  = 30 min, with 2 min pre-bleach and 28 min post-bleach at 1 frame/min) [scale bar  = 50 µm].
(MOV)

Video S4 **Protein synthesis inhibition attenuates axonal recovery of IRES-dependent translation in mCherry^myr^EMCV-eGFP^myr^ mRNA expressing DRG neurons.** Representative FRAP sequence as in Video 3 except cultures were pretreated with 150 µM anisomycin prior to photobleaching (original time lapse  = 30 min, with 2 min pre-bleach and 28 min post-bleach at 1 frame/min) [scale bar  = 50 µm].
(MOV)

Video S5 **Recovery of axonal eGFP fluorescence in mCherry^myr^5′BiP-eGFP^myr^ mRNA expressing DRG neurons.** Representative FRAP sequence of a neuron transfected with pmCherry^myr^5′BiP-eGFP^myr^3′Cal is shown as outlined in Figure 5 with fluorescent intensity shown as a spectrum as indicated (original time lapse  = 30 min, with 2 min pre-bleach and 28 min post-bleach at 1 frame/min) [scale bar  = 50 µm].
(MOV)

Video S6 **Protein synthesis inhibition attenuates axonal recovery of IRES-dependent translation in mCherry^myr^5′BiP-eGFP^myr^ mRNA expressing DRG neurons.** Representative FRAP sequence as in Video 5 except cultures were pretreated with 150 µM anisomycin prior to photobleaching (original time lapse  = 30 min, with 2 min pre-bleach and 28 min post-bleach at 1 frame/min) [scale bar  = 50 µm].
(MOV)
