# Supplementary figures and images for: Localized IRES-Dependent Translation of ER Chaperone Protein mRNA in Sensory Axons
Source: PLoS One. 2012 Jul 24;7(7):e40788. doi: 10.1371/journal.pone.0040788 (PMC3404055; doi:10.1371/journal.pone.0040788)

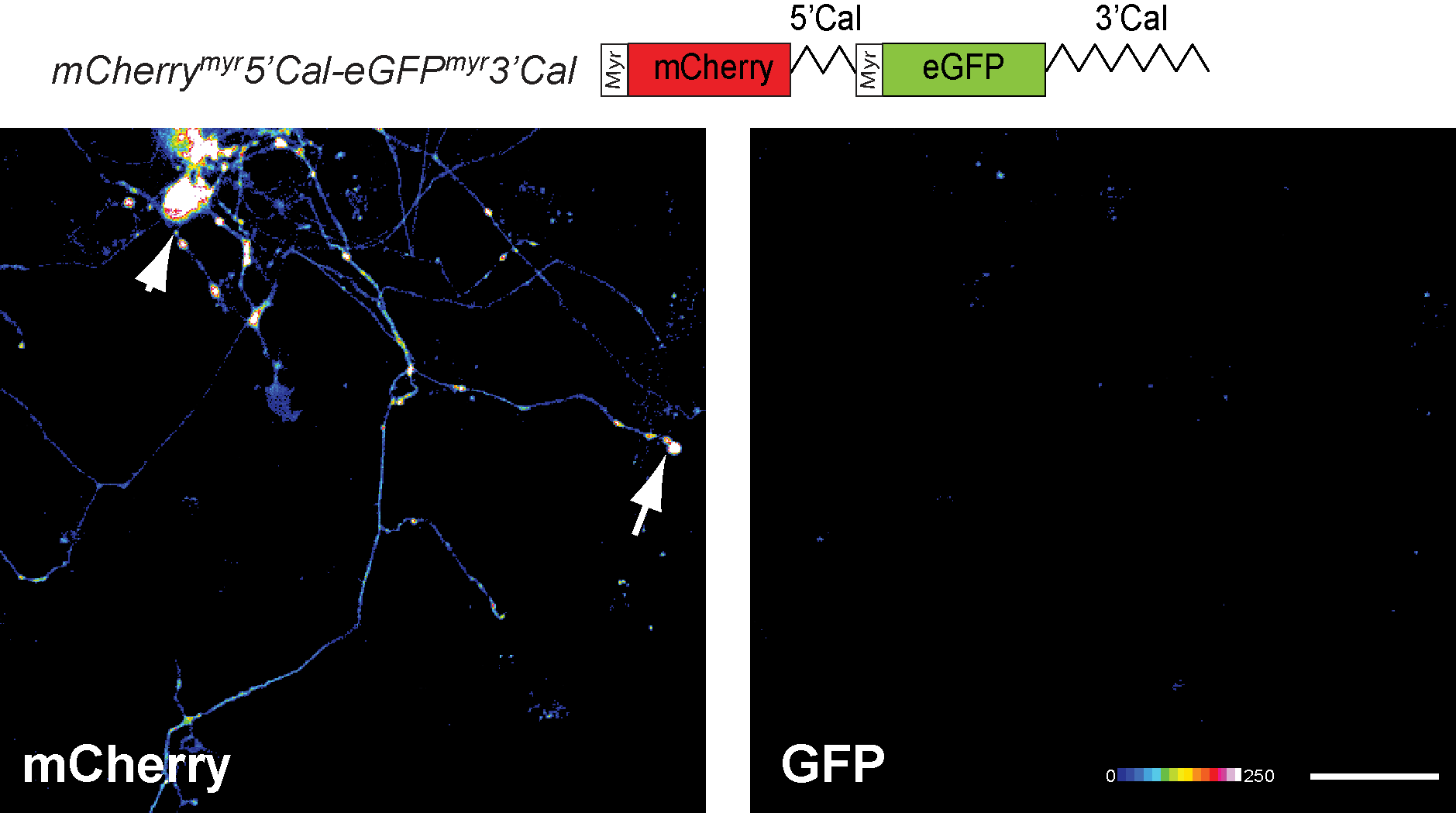

Supplement: Figure S1 — LPA effect in 5′UTR cap-independent translation. Representative images of DRG neurons expressing indicated bicistronic mRNAs are shown after 2 h exposure to 30 µM LPA. Only the mCherry signal is seen for the mCherrymyr5′Cal-eGFPmyr-3′Cal expressing neurons, both in cell body (arrowhead) and axons (arrow). These data indicate LPA does not trigger cap-independent translation through calreticulin’s 5′UTR in sensory neurons [scale bars = 50 µm]. (TIF) [file pone.0040788.s001.tif]
